# Towards Perfect Absorption of Single Layer CVD Graphene in an Optical Resonant Cavity: Challenges and Experimental Achievements

**DOI:** 10.3390/ma15010352

**Published:** 2022-01-04

**Authors:** Abedin Nematpour, Maria Luisa Grilli, Laura Lancellotti, Nicola Lisi

**Affiliations:** 1 Energy Technologies and Renewable Sources Department, Italian National Agency for New Technologies, Energy and Sustainable Economic Development (ENEA), Casaccia Research Centre, Via Anguillarese 301, 00123 Roma, Italy; abedinnemat@grad.kashanu.ac.ir (A.N.); nicola.lisi@enea.it (N.L.); 2 Energy Technologies and Renewable Sources Department, Italian National Agency for New Technologies, Energy and Sustainable Economic Development (ENEA), Portici Research Centre, P.le E. Fermi 1, 80055 Portici, Italy; laura.lancellotti@enea.it

**Keywords:** graphene absorption, Fabry–Perot filter, radio frequency sputtering, CVD graphene

## Abstract

Graphene is emerging as a promising material for the integration in the most common Si platform, capable to convey some of its unique properties to fabricate novel photonic and optoelectronic devices. For many real functions and devices however, graphene absorption is too low and must be enhanced. Among strategies, the use of an optical resonant cavity was recently proposed, and graphene absorption enhancement was demonstrated, both, by theoretical and experimental studies. This paper summarizes our recent progress in graphene absorption enhancement by means of Si/SiO_2_-based Fabry–Perot filters fabricated by radiofrequency sputtering. Simulations and experimental achievements carried out during more than two years of investigations are reported here, detailing the technical expedients that were necessary to increase the single layer CVD graphene absorption first to 39% and then up to 84%. Graphene absorption increased when an asymmetric Fabry–Perot filter was applied rather than a symmetric one, and a further absorption increase was obtained when graphene was embedded in a reflective rather than a transmissive Fabry–Perot filter. Moreover, the effect of the incident angle of the electromagnetic radiation and of the polarization of the light was investigated in the case of the optimized reflective Fabry–Perot filter. Experimental challenges and precautions to avoid evaporation or sputtering induced damage on the graphene layers are described as well, disclosing some experimental procedures that may help other researchers to embed graphene inside PVD grown materials with minimal alterations.

## 1. Introduction

Graphene-based absorbers are receiving a considerable interest due to their potential applications in photovoltaics [1,2,3], as wave modulators [4,5,6,7,8], biological sensors [9,10,11], photodetectors [12,13,14,15], etc. Achieving graphene-based perfect absorbers is quite challenging because single layer graphene has got a weak and spectrally broad absorption of 2.3% over a wideband wavelength range from Vis to FIR (far infrared) [16,17,18,19,20,21].

In the THz and IR regions, however, high quality graphene may show strong interaction with light thanks to generation of surface plasmon polaritons (SPPs), making graphene a promising alternative to typical plasmonic materials [22,23,24,25]. This is thanks to the capability of graphene to support plasmon modes with extremely tight confinement, long lifetime, and low losses at IR and THz frequencies [26,27,28,29].

In addition, tunable graphene electromagnetic response may be achieved by chemical and/or electrical doping or by using a magnetic field [30,31,32].

In the Vis and NIR regions, on the contrary, absence of SSPs makes it necessary to couple graphene with resonant structures such as metamaterials, photonic crystals and plasmonic materials [33,34,35,36,37,38,39].

Many examples of perfect graphene-based absorbers have been reported in the literature basing on the critical coupling concept [40,41,42].

Thongrattanasiri et al. have simulated 100% absorption in doped graphene nanodisks by exploiting the critical coupling conditions [43].

Piper et al. [44] have demonstrated numerically perfect absorption in unpatterned monolayer graphene in the Vis and NIR ranges by means of critical coupling with guided resonances of a photonic crystal slab with a back reflector.

Liu et al. [45] have demonstrated experimentally close to total absorption (85%) in monolayer graphene based on critical coupling with guided resonances in Fano resonance photonic NIR filters, by using a structure with a back reflector.

Xiao et al. [46] have reported about a general theoretical method for tailoring the absorption bandwidth of graphene via critical coupling in the NIR range by using a simple two-port resonant structure composed of a graphene-covered two-dimensional photonic crystal slab.

Jin et al. [47] have reviewed recent advances of graphene-based architectures for perfect absorption from Vis to THz band, both narrowband and broadband, and have discussed also about criticalities in the practical implementation of the simulated structures.

Another strategy to enhance graphene absorption is to exploit the electric field enhancement by resonant cavities [48,49,50]. Table 1 reports the graphene absorption values obtained by many authors by exploiting the electric field enhancement inside Fabry–Perot resonant cavities.

Nulli et al. reported theoretically that the optical absorption of single-layer graphene can be enhanced up to 50% by increasing the electric field on the surface of undoped graphene by placing graphene inside symmetric Fabry–Perot structures [48].

Ferreira et al. have calculated the same absorption result for single layer graphene by utilizing one Fabry–Perot cavity and they have simulated also that graphene optical absorption can increase up to 100% using two symmetric Fabry–Perot cavities [49].

Zand et al. have used a genetic optimization algorithm coupled to a transfer matrix code to design one-dimensional aperiodic multilayer microstructure embedding single layer graphene, where near-total absorptions at selected wavelengths is obtained by existence of critical coupling [61]. They simulated that, compared with asymmetric Fabry–Perot-based designs, aperiodic structures may provide higher efficiency for the spatial selective localization of the resonant modes.

Furchi et al. [63] have demonstrated experimentally in 2012 a graphene-based photodetector. Absorption enhancement up to 60% at 855 nm was demonstrated by inserting exfoliated μm-sized graphene flakes inside a dielectric multilayer stack grown by combining plasma-enhanced chemical vapor deposition and molecular beam epitaxy.

Many of the theoretical studies found in the literature simulating perfect graphene absorption consider for single layer graphene a very high mobility µ of about 10,000 cm^2^/Vs, which is generally much higher than the real one (non-isolated from the environment, CVD graphene), leading sometimes to too overoptimistic predictions [64,65].

Apart from the work from Furchi et al., the enhancement of graphene absorption by using an optical resonant cavity has been rarely explored experimentally due to the many experimental challenges related to embedding graphene inside metals or dielectric stacks [63,66].

The graphene absorption enhancement inside an optical resonant cavity is generally narrowband and is, therefore, more suitable for application in photodetectors, sensors, or absorption filters [63,66,67].

In the present work, we summarize our recent studies [67,68,69] on CVD graphene absorption inside three different Fabry–Perot (FP) filters fabricated by radiofrequency sputtering. Experimental challenges related to graphene-based Fabry–Perot filters fabrication are described in detail with the aim to provide a useful recipe which can help researchers to embed graphene inside materials grown by conventional physical vapor deposition (PVD) techniques, without altering its properties.

In the optimized structure, a high absorption of 84% at 3150 nm was measured [67] in case of large area (1 inch) single layer CVD graphene, which is the highest value of absorption so far experimentally achieved for single layer graphene inside a Fabry–Perot optical cavity.

## 2. Materials and Methods

### 2.1. Fabry–Perot Filter: Simulation

**Figure 1:**
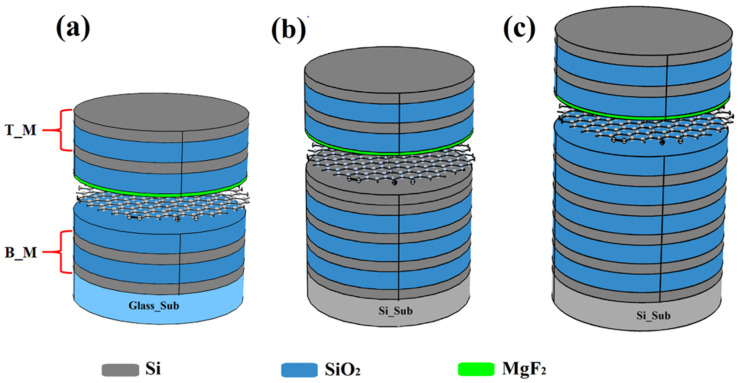
Scheme of the Fabry–Perot filters embedding SLG: (**a**) symmetric FP (FP1), (**b**) asymmetric FP (FP2) and (**c**) asymmetric reflective FP (FP3). T_M and B_M stand for top and bottom mirrors.

**Figure 2:**
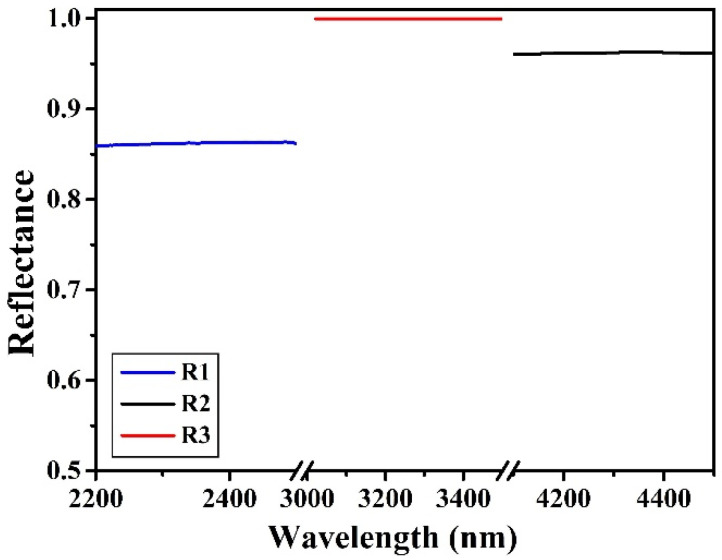
Simulated reflectance curves of the bottom mirrors shown in Figure 1. R1, R2, and R3 are the reflectance of bottom mirrors of FP1, FP2, and FP3, respectively.

A Fabry–Perot optical filter consists of a cavity separated by two flat and parallel high reflecting mirrors where light experiences multiple reflections. Simulation of FP filters was carried out by using the Wave Optics Module of Comsol Multiphysics software. Our Fabry–Perot filters consisted of a sequence of alternate quarter wave thick high refractive index Si (H) and low refractive index SiO_2_ (L) layers, for use in the near to mid IR. Three different Fabry–Perot filters were simulated: (i) a symmetric FP filter with identical top and bottom mirrors (FP1), (ii) an asymmetric FP filter where the bottom mirror had a higher reflectance due to a larger number of layers with respect to the top mirror (FP2), and (iii) an asymmetric reflective FP filter obtained by further increasing the bottom mirror layers (FP3). FP1 and FP2 filters worked in transmissive mode, while FP3 worked in reflective mode, due to the high reflectance of the bottom mirror (99.7%). The structure of FP1, FP2, and FP3 filters was the following: (i) air/HLH LL HLH/sub, (ii) air/HLHL HH LHLHLH/sub, and (iii) air/HLH LL HLHLHLHLH/sub, respectively. For FP1 and FP3 the cavity was (LL), i.e., constituted by a half wavelength thick SiO_2_ layer, for FP2 the cavity (HH) was made by a half wavelength thick Si layer. This last FP structure, a bit different from the other two, was chosen because of its particular distribution of the electric field characterized by two maxima. A single layer graphene (SLG) was embedded inside the FP structure and located at the position where the electric field was maximum to enhance its absorption. SLG was covered by a 30 nm thick MgF_2_ layer, meant to protect graphene during the later sputtering deposition. Figure 1 shows the three FP filters embedding SLG, and Figure 2 shows the simulated reflectance of the bottom mirrors in the three cases, R1, R2, and R3. R1 is also the reflectance of the top mirror, being identical for the three filters.

**Figure 3:**
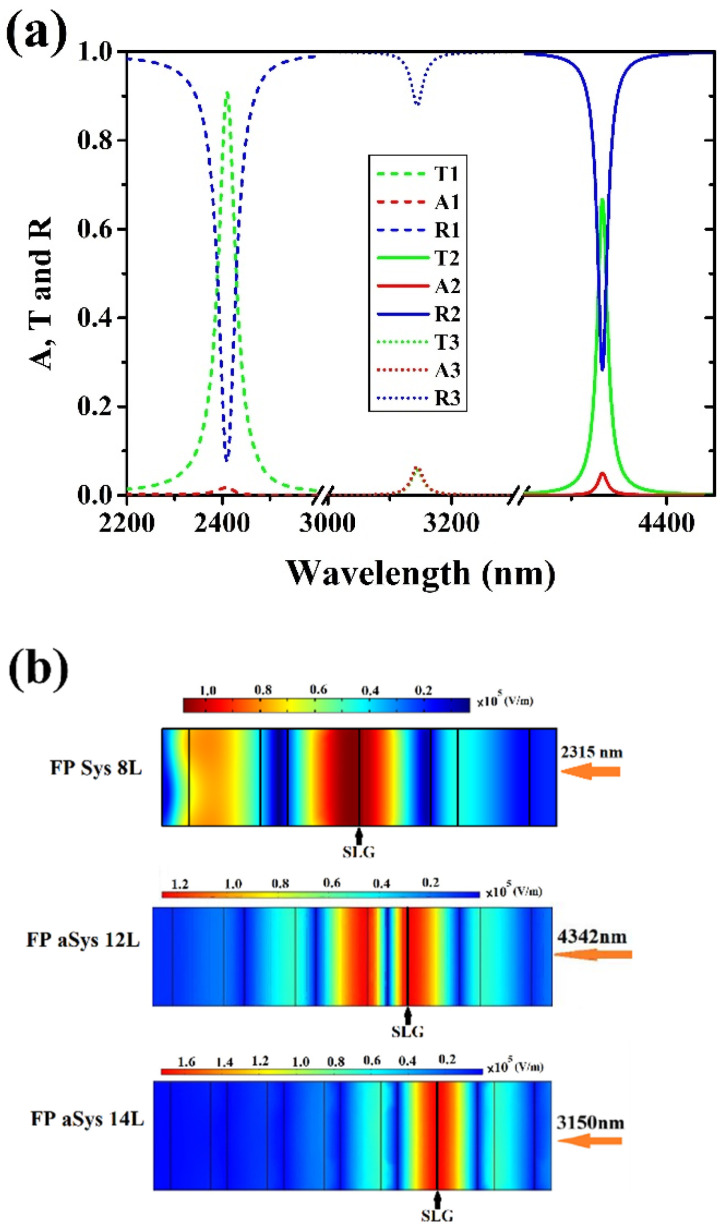
(**a**) Simulated transmittance (T), reflectance (R), and absorption (A) of Fabry–Perot filters FP1, FP2, and FP3; (**b**) electric field distribution inside the Fabry–Perot filters. SLG position is indicated in the three filters by a black line positioned at electric field maximum (red color).

Figure 3a shows the simulated transmittance (T), reflectance (R), and absorption (A) of the FP1, FP2, and FP3 filters, centered at λ = 2315, 4342, and 3150 nm, respectively. Figure 3b shows the electric field distribution inside the filters. The simulation has been carried out in case of a TE polarized light at normal incident angle.

Table 2 reports the simulated and experimental absorption values of SLG embedded in the three FP filters and the value of electric field (E_G_) where SLG is positioned. The central wavelength of the Fabry–Perot filters is different for the three cases because it results from different experiments, however, central wavelength position in the considered wavelength range, only causes small variations of T, R, and A due to the wavelength dependence of Si and SiO_2_ optical constants. As we can observe from Table 2, the highest graphene absorption is obtained in the structure where the electric field is maximum.

### 2.2. Fabry–Perot Filter: Fabrication

The Fabry–Perot filters consisted of alternate layers of Si and SiO_2_ with optical thickness equal to a quarter wavelength (λ/4, where λ is the central wavelength of the FP filter). Si and SiO_2_ layers were fabricated by radio frequency sputtering in MRC (Material Research Corporation) systems, starting from 99.999% purity Si and/or SiO_2_ targets, as described elsewhere [67,68,69]. For FP1 and FP2, two different sputtering conditions were chosen: in case of all the layers except for the first 30 nm of the SiO_2_ layer covering graphene, a radiofrequency sputtering of 200 W and a sputtering pressure of 1 Pa were used. For the first 30 nm of SiO_2_ layer deposited onto graphene, a milder sputtering condition was used: i.e., sputtering power of 50 W and sputtering pressure of 3 Pa. For FP3 filter, grown in a different system, the same sputtering conditions (sputtering power of 200 W and sputtering pressure of 0.53 Pa) were used for Si and all the SiO_2_ layers, due to the fact that such system geometry allowed a milder sputtering process.

MgF_2_ layer was carefully evaporated in a Balzer BAE 250 evaporation system, and the evaporation rate was controlled by a quartz crystal microbalance.

### 2.3. CVD Graphene: Fabrication and Transfer

Single layer graphene was grown by CVD on Cu foils previously submitted to a pre-oxidation treatment at 250 °C for 90 min. CVD growth conditions were the following: substrate temperature 1070 °C, 10 mbar pressure by using 0.025 SCCM ethanol vapor with 20 SCCM Ar and 10 SCCM H_2_. After the growth, graphene was removed from the back of the Cu substrate by means of an oxygen plasma cleaner (100 W, 4 min). For graphene transfer, a procedure reported elsewhere was applied [70]. Summarizing: a few drops of cyclododecane (20% solution in dichloromethane) were spin coated on top of graphene, and the Cu foils were left floating on an ammonium persulfate (PSA) water solution (120 g/L) for 3 h at 4 °C, until complete copper etching occurred. Afterword, the sample was rinsed in distilled water and scooped directly with the bottom multilayer, then submitted to a heating treatment @70 °C for a few hours and to ethyl acetate vapor cleaning to remove cyclododecane residues. The same transfer procedure was applied for transferring SLG onto other kinds of substrates (Si or quartz) used for graphene characterization.

### 2.4. Embedding Graphene Inside the Fabry–Perot Filters

**Figure 4:**
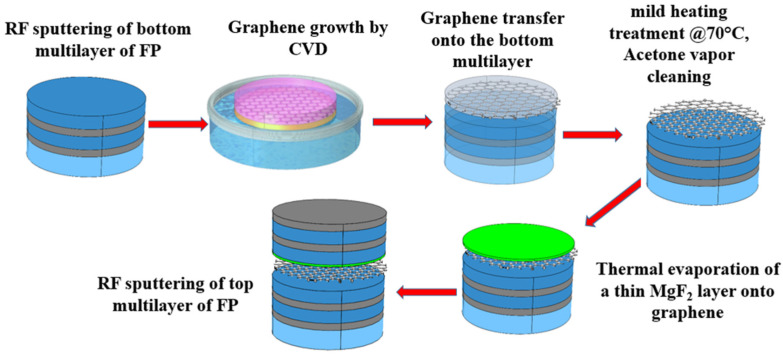
Graphene-based Fabry–Perot fabrication steps in case of the symmetric Fabry–Perot FP1.

Figure 4 shows the experimental steps used for the insertion of SLG inside the FP filters. In our experiments, to maximize absorption, graphene was always positioned inside the multilayer where the electric field was maximum [48,57]. In the symmetric FP1 filter, graphene was positioned in the middle of the cavity (LL), i.e., sandwiched between the two SiO_2_ layers which separate the top and bottom mirrors. In the asymmetric FP2 filter, two maxima of electric field occurred, and graphene was positioned onto the upper Si layer of the cavity (HH). In the asymmetric reflective FP3 filter, graphene was positioned in the middle of the cavity (LL), i.e., sandwiched between the two SiO_2_ layers.

The fabrication procedure of the multilayer structure was the following. First, the bottom multilayer structure and two CVD graphene layers were fabricated separately in the sputtering and CVD chambers, respectively. For each sputtering deposition run, two substrates (quartz or Si) were loaded in the chamber, one for the reference and the other for the graphene-based FP filter. Similarly, two graphene layers were prepared in the same run, one for insertion into the FP filter and the other for characterizations. Then, the graphene layer was transferred on the top of the bottom multilayer by the mild transfer process described above. The multilayer structure topped by graphene and the reference multilayer (without graphene) were transferred into the evaporation chamber for the growth of 30 nm MgF_2_ protective layer. After the evaporation of the MgF_2_ layer, the two samples were transferred back into the sputtering chamber for the deposition of the top multilayer structure.

### 2.5. Materials Characterizations

Transmittance and reflectance measurements in the NIR were carried out with a PerkinElmer 900 spectrophotometer, while MIR measurements were performed with a high resolution FTIR Perkin Elmer instrument. The reflectance measurements were obtained by using calibrated standards, i.e., an Ocean Optics Al mirror certified in the range 250–2500 nm in the first case and an Al infrared reflectance standard with a SiO overcoat certified by the National Physics Laboratory (NPL) in the wavenumber range of 4000–200 cm^−1^ in the latter case. For FTIR measurements, a Fixed-Angle Specular Reflectance Accessory from Perkin Elmer was used.

The Raman measurements were carried with a Renishaw inVia Reflex Raman spectrometer using a 514.5 nm excitation source.

## 3. Results and Discussion

### 3.1. Graphene Absorption Simulation

**Figure 5:**
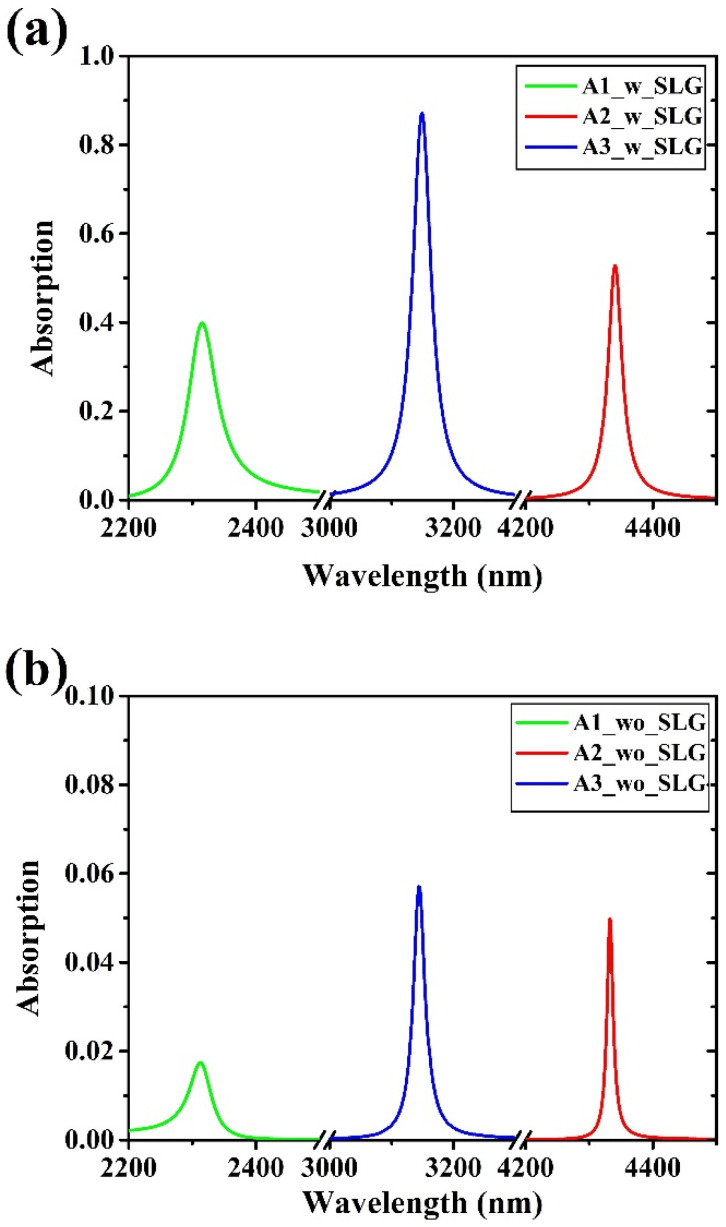
(**a**) Simulated absorption curves of SLG inside the Fabry–Perot filters in case of symmetric FP1 (A1), asymmetric FP2 (A2), and asymmetric reflective FP3 (A3). (**b**) Simulated absorption of FP filters without graphene insertion.

Figure 5a shows the simulated absorption of graphene inside the three FP filters while Figure 5b shows the FPs’ absorption without the single layer graphene. The wavelength-dependent absorption A(λ) was inferred from the wavelength-dependent transmittance T(λ) and reflectance R(λ) (A(λ) = 1 − R(λ) − T(λ)) [71,72], by using the Wave Optics Module of Comsol Multiphysics software. Graphene optical properties were simulated by a wavelength-dependent complex refractive index $n_{g}(\omega )=\sqrt{\varepsilon_{g}(\omega )}$ [73,74], where the single-layer graphene permittivity was [75,76,77]:

$$\varepsilon_{g}(\omega )=\varepsilon_{b}+\frac{i\sigma (\omega )}{t_{G}\omega \varepsilon_{0}}\;$$

being $\varepsilon_{b}$(2.5) [78,79], *t*_G_ (0.35 nm) [80,81], and *σ*(*ω*) the intrinsic contribution to the graphene relative permittivity, the thickness of single layer graphene and the graphene optical conductivity, respectively.

The graphene optical conductivity was considered as the sum of interband transitions and Drude-like intraband conductivity [82,83,84,85]:

$$\begin{array}{l} \sigma (\omega )=\frac{\sigma_{0}}{2}[\tanh(\frac{\hbar \omega +2E_{f}}{4k_{B}T})+\tanh(\frac{\hbar \omega -2E_{f}}{4k_{B}T})]\\ -\frac{i\sigma_{0}}{2\pi }\log(\frac{{\left(\hbar \omega +2E_{f}\right)}^{2}}{{\left(\hbar \omega -2E_{f}\right)}^{2}+{\left(2k_{B}T\right)}^{2}})\\ +i\frac{4\sigma_{0}}{\pi }\frac{E_{f}}{\hbar \omega +i\hbar \gamma } \end{array}$$

with $\sigma_{0}$ = (e24ℏ) [82,86], $E_{f}$ the Fermi energy, and γ the intraband scattering rate. In the simulation, ℏγ = 40 meV [83] and $E_{f}$ = 190 meV corresponding to slightly doped graphene as reported elsewhere [83,87].

**Figure 6:**
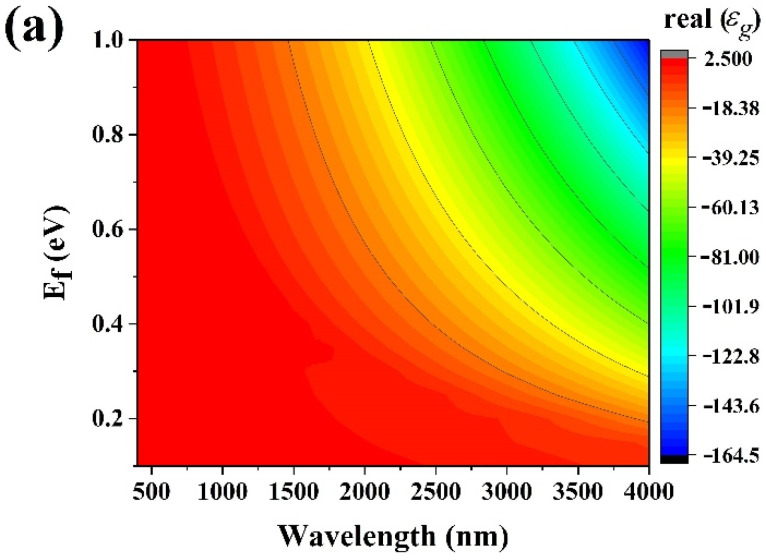

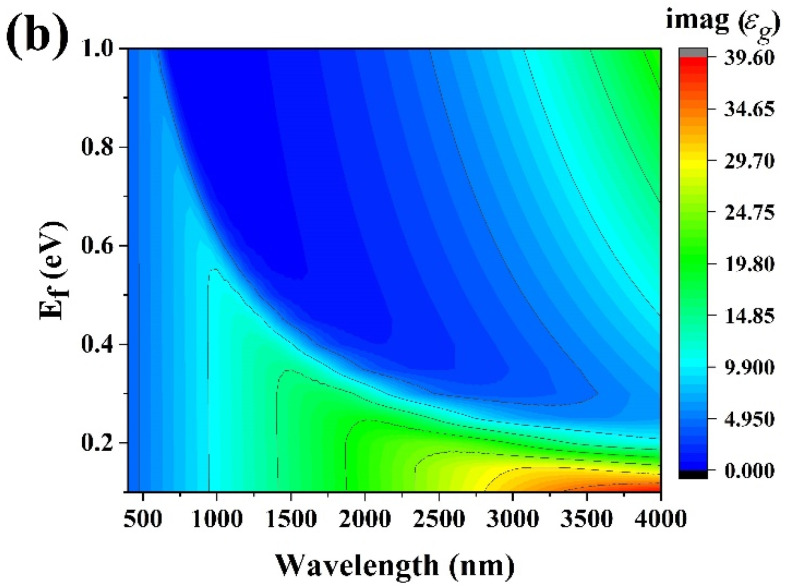
Simulated SLG permittivity as a function of wavelength and Fermi level. (**a**) Real and (**b**) imaginary parts.

Single layer graphene permittivity was simulated also as a function of the wavelength and Fermi level, as shown in Figure 6.

### 3.2. Graphene Quality Assessment

The quality of graphene was checked after each experimental step, i.e., after MgF_2_ evaporation, after SiO_2_ sputtering on MgF_2_/graphene layers and after the whole filter fabrication process.

**Figure 7:**
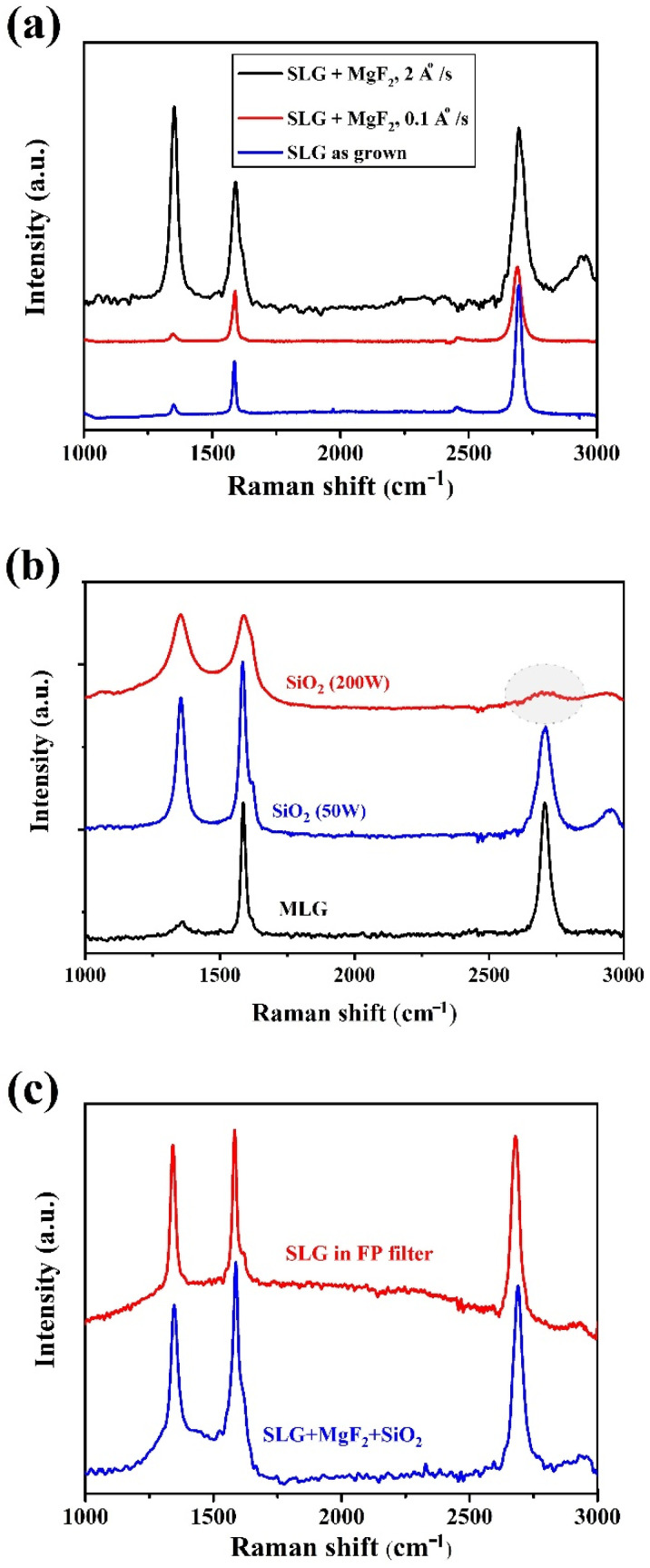

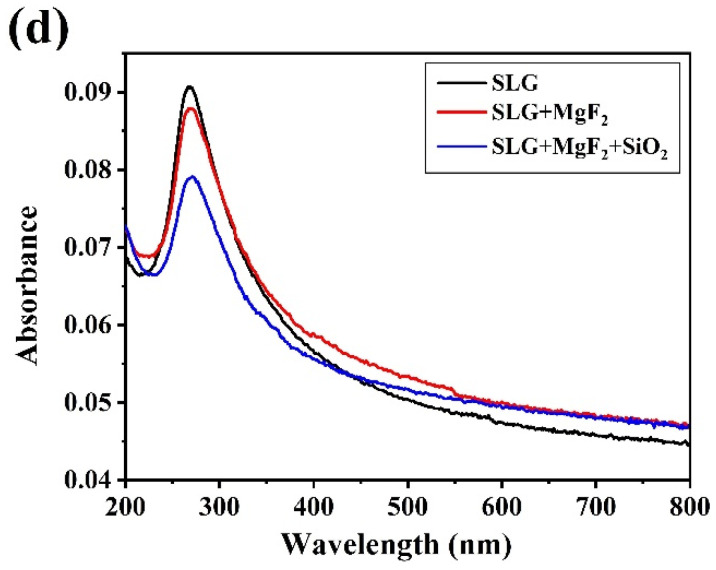
Raman spectra of: (**a**) SLG showing the effect of MgF_2_ evaporation rate, (**b**) MLG showing the effect of SiO_2_ sputtering conditions, and (**c**) SLG showing the effect of insertion inside the Fabry–Perot filter. (**d**) Comparison of absorbance of SLG pristine (as transferred on quartz substrate) and after MgF_2_ evaporation and SiO_2_ sputtering.

Initially, for the growth of the first 30 nm of SiO_2_ layer directly onto SLG, a mild sputtering condition was applied by increasing the sputtering pressure and decreasing the radiofrequency power. Notwithstanding, graphene damage could not be avoided, as demonstrated by Raman analysis (not shown here), and a further graphene protection was necessary. Therefore, to avoid the sputtering of the SiO_2_ layer directly onto graphene, a thin protective MgF_2_ layer was evaporated prior to the sputtering of SiO_2_. We have chosen MgF_2_ due to its refractive index and extinction coefficient values, very close to those of SiO_2_. This is necessary since MgF_2_ becomes part of L (the low index layer) of the multilayer stack. Other fluoride materials could have served the purpose. Furthermore, the evaporation of the MgF_2_ layer required some care since we observed that if evaporation occurred at a high rate, graphene defects band D increased. Figure 7a compares the Raman spectra of pristine graphene and graphene after evaporation of MgF_2_ at high (2 Å/s) and low (0.1 Å/s) evaporation rates. Only the latter case left graphene unchanged. The effect of deposition rate of SiO_2_ was preliminarily evaluated in experiments with ML graphene with unoptimized MgF_2_ deposition (curve of Figure 7b). The effect of the optimized (low power and high pressure) SiO_2_ deposition on SL graphene can be seen in the red and blue curves of Figure 7c. Here the comparison of the red (Raman from the back of the peeled stack) and blue curves (Raman from the top through the SiO_2_), suggests that some extent of the modifications of the D and 2D bands by SiO_2_ is not due to lattice damage but to optical and electronic “proximity” influences on graphene by the stack.

Despite the increase of the defectiveness D band, the optical properties of graphene were preserved, as shown by the comparison of UV-Vis absorbance curves of pristine graphene transferred on a quartz substrate and graphene after MgF_2_ and SiO_2_ deposition (Figure 7d). Moreover, two-point probe measurements of SLG resistance prior and after the MgF_2_ evaporation and the SiO_2_ sputtering confirmed that graphene resistance was almost unaffected by these processes. SLG sheet resistance increased, in fact, of less than 10% with respect to the initial value of about 800 Ω/sq.

The results obtained after several attempts to not damage graphene can be summarized as following: (i) a thin MgF_2_ layer is essential to protect graphene, (ii) evaporation of the thin MgF_2_ layer should occur with a very low rate, (iii) the presence of the thin MgF_2_ layer is not by itself sufficient to avoid the damage if the sputtering process is too energetic, (iv) the combination of evaporated MgF_2_ and mild SiO_2_ sputtering increases the graphene D band but preserves the optical and electrical properties of pristine graphene, finally (v) we cannot certainly assign the measured D band increase to an effective graphene “damage” while inside the stack due to possible proximity effects by the embedding layers on the Raman spectrum itself.

### 3.3. Graphene Absorption Inside the Fabry Perot Filters: Experimental

**Figure 8:**
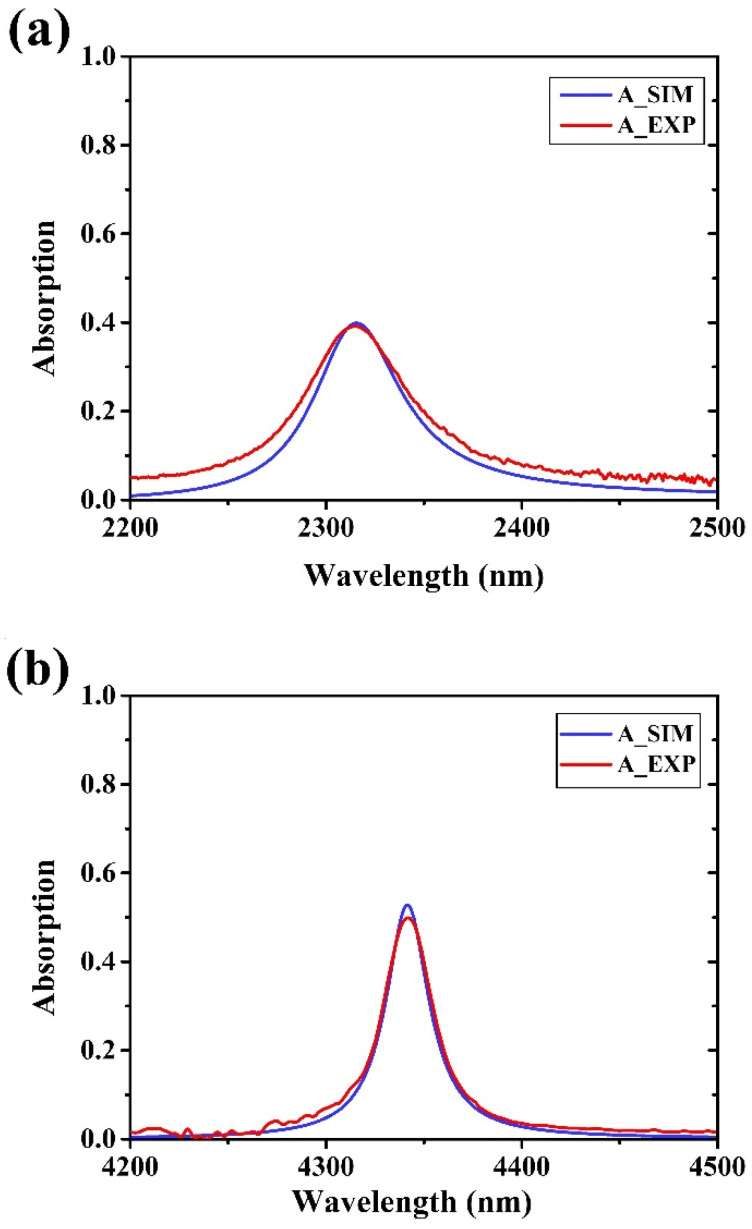

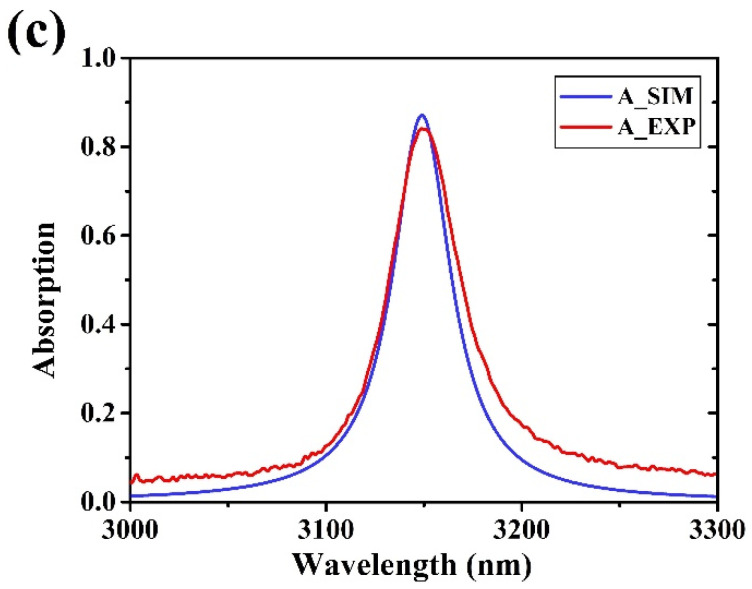
Comparison between simulated and experimental absorption curves of single layer graphene inside the Fabry–Perot filters: (**a**) symmetric FP1, (**b**) asymmetric FP2, and (**c**) asymmetric reflective FP3.

Figure 8a–c shows the comparison between the simulated and measured total absorption curves of graphene inside the three Fabry–Perot filters. The total absorption values (Table 2) include, also, the contribution of SLG absorption (2.3%) and of the FP materials (Figure 5b). A very good agreement between the simulated and measured absorption values was obtained, and only some broadening of the experimental curves was found in all cases. As it can be noticed, the experimental graphene absorption trend is to increase when passing from a symmetric structure to an asymmetric one and it increases further by increasing the reflectance of the bottom mirror. Even though a comparison of the absolute absorption values is not possible due to the different FPs’ central wavelengths, the absorption trend strongly suggests that inside a Fabry–Perot cavity a high graphene absorption may be obtained only in asymmetric reflective structures. In our experiments, for example graphene absorption values increased from 39 to 84% when passing from a symmetric transmissive to an asymmetric reflective structure. The highest absorption value obtained in the asymmetric reflective FP3 filter is due to the fact that the incident light inside the cavity is reflected a greater number of times which allows to graphene a higher number of multiple absorptions. The number of reflections is in fact related to the Fabry–Perot finesse which is defined by [68,88,89,90]:

$$F_{s}=\frac{\pi \sqrt{R}}{1-R}\;\mathrm{and}\;F_{as}=\frac{\pi \sqrt[{4}]{R_{Top}R_{Bottom}}}{1-\sqrt{R_{Top}R_{Bottom}}}$$

Above, the left-hand side formula represents the finesse of the symmetric FP filter (FP1), where *R* is the reflectance of the top and bottom mirrors, while on the right-hand is the finesse of the asymmetric filters (FP2 and FP3). In our FP filters, the finesse increased from 21 to 31.5 when passing from the symmetric structure of Figure 1a (FP1) to the asymmetric reflective structure of Figure 1c (FP3).

Absorption of unpatterned and undoped single layer graphene up to 84% has been never reported experimentally in a Fabry–Perot filter. Comparable or even higher absorption values have instead been obtained by other authors by using graphene coupled to non-planar resonant plasmonic structures [45,91,92].

Enhancing the light-matter interaction in single-layer graphene by the use of optical microcavity requires no stringent constrains on the graphene electrical properties, such as the sheet resistance, and may find application in a variety of graphene-based devices, such as optical absorption modulators, light emitters, and optical attenuators, and provides new routes to graphene photonics for applications in spectroscopy, communications, sensing, and security.

Results, validated here at NIR and MIR wavelengths, may be applied in principle at different wavelengths ranging from Vis to THz by properly choosing the materials of the layers and of the substrate. Moreover, as obtained by our previous simulations, modification of the filter amplitude and bandwidth may be achieved by: (i) using multilayer graphene instead of single layer graphene [68], (ii) using two or more SLGs positioned in the filter where the electric field has its maxima [69], and (iii) increasing the number of cavities of the multilayer structure [68].

**Figure 9:**
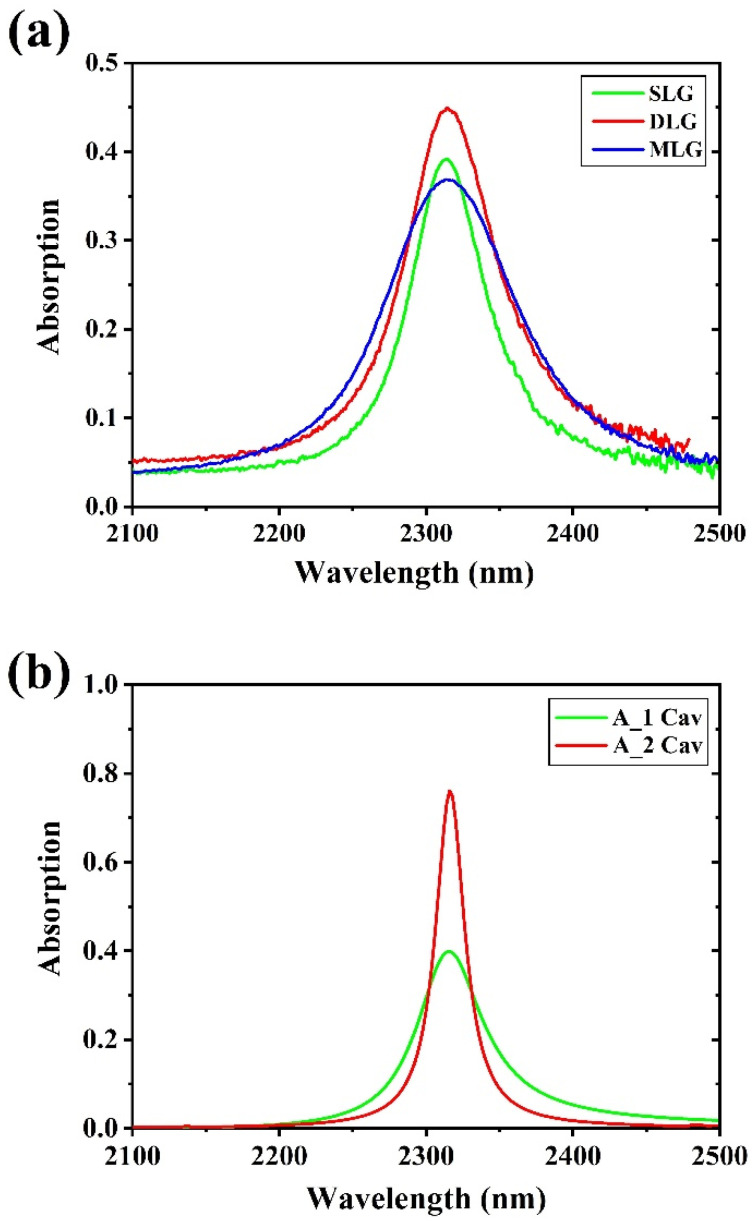
Absorption of graphene layers inside the FP1 as a function of number of graphene layers (**a**) and absorption of a single layer graphene in a single Fabry–Perot cavity and in a dual Fabry–Perot cavity (**b**).

Figure 9a shows, as an example, the simulated absorption of a single layer graphene, of a double-layers graphene (DLG) and of a five-layers graphene (MLG) embedded in the symmetric FP1. As it can be noticed, absorption bandwidth increases by increasing the number of layers, while absorption amplitude increases in DLG and decreases in MLG. The latter behavior can be attributed to the higher intrinsic absorption of MLG which reduces the reflections of the light inside the optical cavity, and to the fact that MLG perturbs largely the optical quality of the Fabry–Perot cavity [68].

Figure 9b shows the effect of the number of Fabry–Perot cavities on the absorption of a single layer graphene [68], showing a higher graphene absorption inside a dual cavity Fabry–Perot filter.

Results are in accordance with what has been reported by other authors. In [46], Xiao et al. have numerically modeled, in a structure different from ours, the bandwidth absorption as a function of the number of graphene layers finding a broader absorption band by increasing the number of graphene layers from 1 to 7. In [49], Ferreira et al. have numerically simulated that absorption of single layer graphene increases by using a dual cavity Fabry–Perot filter instead of a single cavity one.

**Figure 10:**
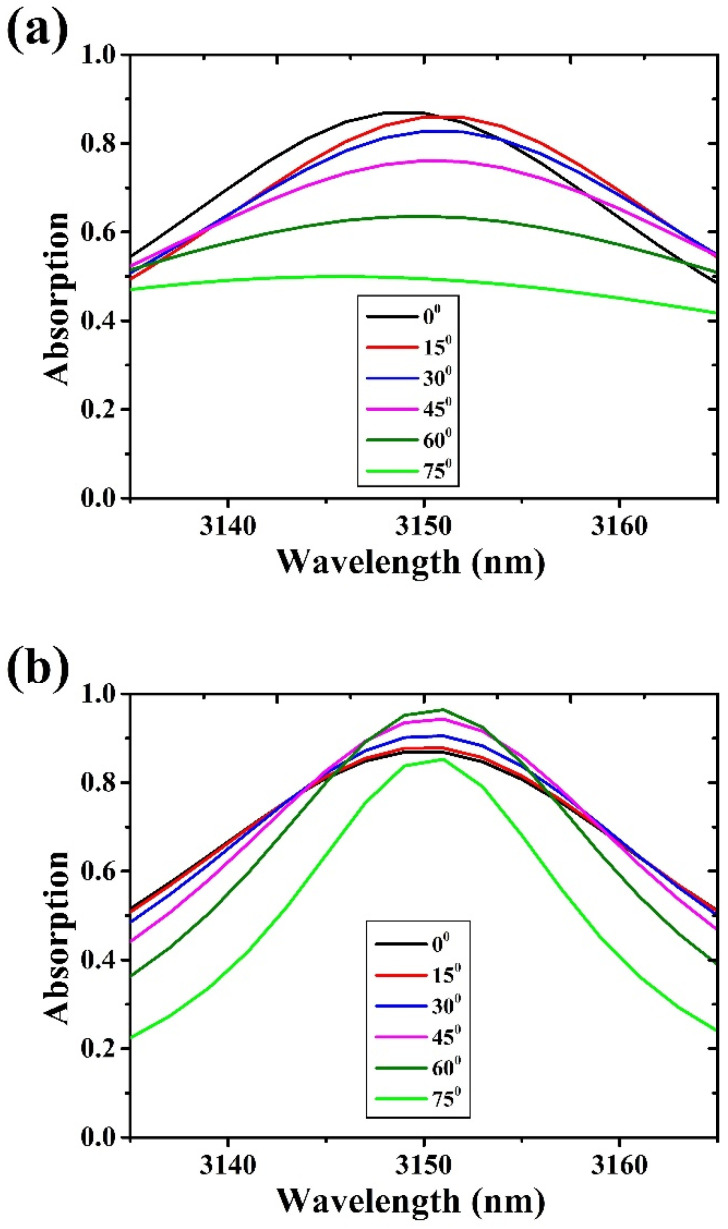
SLG optical absorption as a function of wavelength for different incident angles at (**a**) TE and (**b**) TM polarization.

Graphene absorption discussed so far was obtained in the case of a TE polarization. For the optimized FP structure, i.e., the FP3 filter, the simulation was carried out, also, in case of TM polarization. Figure 10 shows the comparison between the simulated absorption of SLG for TE and TM polarizations as a function of the incident angle. As it can be noticed, in case of TE polarization the absorption maximum is reached for incident radiation, while for TM polarization it occurs at a higher incident angle of 60°.

## 4. Conclusions

This work summarizes our recent findings on single layer graphene absorption when embedded in dielectric multilayer stacks. The increase of single layer graphene absorption obtained by exploiting the electric field enhancement inside a resonant optical cavity was modeled and experimentally demonstrated in three different Fabry–Perot filters with central wavelengths varying in the NIR-MIR spectral ranges. The Fabry–Perot filters were fabricated by radiofrequency sputtering, and consisted of alternate quarter wave thick Si and SiO_2_ layers. Results demonstrated that graphene absorption greatly increases when graphene is embedded inside an asymmetric Fabry–Perot structure, reaching its maximum in case of a reflective Fabry–Perot filter. SLG properties were preserved during the sputtering process by applying a thin, slowly evaporated MgF_2_ layer, allowing the effective embedding of graphene inside thick monolithic structures fabricated by conventional PVD techniques. Such a high graphene absorption discloses exciting potentiality for exploitation of 2D materials in new optoelectronic devices for application in the NIR-MIR spectral range.

## Figures and Tables

**Table 1:** Summary of simulated and experimental absorption of graphene inside Fabry–Perot structures in the Vis-THz region, as reported in the literature.

Authors	Wavelength	Absorption	Simulation	Experiment	Ref.
Nulli et al.	Wavelength regardless	0.5	Yes	No	[48]
Ferreira et al.	Wavelength regardless	~1	Yes	No	[49]
Xu et al.	Telecommunication wavelengths	0.5	Yes	No	[51]
Shuhan Chen	1600 nm	0.889	Yes	No	[52]
Yu et al.	550 nm	0.995	Yes	No	[53]
Wei et al.	1537 and 1579 nm	0.995	Yes	No	[54]
Bian et al.	2.5146 THz	0.997	Yes	No	[55]
Vasić et al.	Near-infrared to Terahertz	~1	Yes	No	[56]
Deng et al.	THz range	~1	Yes	No	[57]
Zheng et al.	600 nm	~1	Yes	No	[58]
Bian et al.	THz range	~1	Yes	No	[59]
Doukas et al.	1550 nm	~1	Yes	No	[60]
Zand et al.	1200, 1550 and 1900 nm	~1	Yes	No	[61]
Chen et al.	THz range	~1	Yes	No	[62]
Furchi et al.	855 nm	0.6	Yes	Yes	[63]

**Table 2:** Graphene-based Fabry–Perot characteristics. Simulated and experimental absorption and electric field value at SLG position (maximum of electric field).

	Wavelength (nm)	A_SIM (%)	A_EXP (%)	E_G_ (V/m)
FP1	2315	40	39	106,604
FP2	4342	53	50	125,941
FP3	3150	87	84	172,663

## Data Availability

Data are available on request from the corresponding author.
